# Increasing Children’s physical Activity by Policy (CAP) in preschools within the Stockholm region: study protocol for a pragmatic cluster-randomized controlled trial

**DOI:** 10.1186/s13063-022-06513-4

**Published:** 2022-07-19

**Authors:** C. Chen, V. H. Ahlqvist, P. Henriksson, J. H. Migueles, F. Christiansen, M. R. Galanti, D. Berglind

**Affiliations:** 1 Department of Global Public Health, Karolinska Institutet, Solna, Sweden; 2 Centre for Epidemiology and Community Medicine (CES), Region Stockholm, Stockholm, Sweden; 3 Department of Health, Medicine and Caring Sciences, Linköping University, Linköping, Sweden; 4 PROFITH “PROmoting FITness and Health through physical activity” Research Group, Sport and Health University Research Institute (iMUDS), Department of Physical Education and Sports, Faculty of Sport Sciences, University of Granada, Granada, Spain

**Keywords:** Physical activity, Policy, Intervention, Cluster-randomized controlled trial, Preschool, Accelerometer-based measurement

## Abstract

**Background:**

Systematic reviews suggest that preschool environmental/organizational changes may be effective in increasing physical activity (PA) levels of preschool children, but evidence is scarce regarding feasible, effective, and equitable interventions that can be scaled up. Specifically, it is essential to understand whether introducing a multicomponent organizational change in terms of policy in the preschool context may be beneficial for children’s PA levels and concomitant health outcomes. To bridge this knowledge gap, our main aim is to examine the feasibility and effectiveness of a policy package in increasing PA levels in preschool children, using a large-scale pragmatic cluster-randomized controlled trial.

**Methods:**

This proposed study is a pragmatic cluster-randomized controlled trial with two conditions (intervention and control with a 1:1 ratio) with preschools as clusters and the unit of randomization. We aim to recruit approximately 4000 3–5-year-old children from 90 preschools and retain more than 2800 children from 85 preschools to provide adequate statistical power for the analyses. The intervention to implement is a co-created, multicomponent policy package running for 6 months in preschools randomized to intervention. Change in accelerometer measured PA levels in children between intervention and control from pre- and post-intervention will be the primary outcome of the study, while secondary outcomes include health outcomes such as musculoskeletal fitness, psychosocial functioning, and absence due to illness in children among others. Implementation will be studied carefully using both quantitative (dose, fidelity) and qualitative (interview) methodologies. The change in primary and secondary outcomes, from pre- to post-intervention, will be analyzed with linear mixed-effect models (to allow both fixed and random effects) nested on a preschool level.

**Discussion:**

This is a large-scale co-creation project involving the City of Stockholm, childcare stakeholders, preschool staff, and the research group with the potential to influence more than 30,000 preschool children within the Stockholm area. The study will add reliable evidence for the implementation of PA policies at the organizational level of preschools and clarify its potential effect on objectively measured PA and health markers in children.

**Trial registration:**

ClinicalTrials.govNCT04569578. Prospectively registered on September 20, 2020.

**Supplementary Information:**

The online version contains supplementary material available at 10.1186/s13063-022-06513-4.

## Background

Low levels of physical activity (PA) are a growing global concern [1] and efforts to increase PA are imperative for children of preschool age [2]. Total PA and moderate to vigorous PA (MVPA) are positively associated with the healthy development of young children (< 5 years) [3]. For example, childhood PA is positively associated with musculoskeletal fitness, cardiometabolic health, and psychosocial health [3]. In contrast, excessive sedentary time (ST) in youth is associated with increases in markers of cardiometabolic disease risk [4]. Consistent data show that low levels of musculoskeletal fitness in children are longitudinally associated with low bone mass density, adiposity, metabolic risk factors, and premature mortality [5]. In addition, the healthy PA habits developed during early ages may track to later stages of life [6, 7]. Consequently, the assessment of PA and musculoskeletal fitness in youth is relevant from a public health perspective.

Despite the known benefits of PA, children in general engage in low levels of PA and their PA tends to decrease with age [8]. A recent Swedish accelerometer-based national survey showed that only 23% of adolescent girls and 43% of boys reached the recommended level of a minimum of 60 min of daily MVPA [9]. Moreover, low accelerometer measured PA levels have been shown in preschoolers, as highlighted by a study of approximately 1000 Swedish preschool children [10].

Higher intensity PA (e.g., MVPA) is a major determinant of physical fitness in children [11]. In addition to the observed low levels of PA among preschoolers, physical fitness among youths is declining globally [12] and in Sweden [13]. Notably, the decline in fitness level is more pronounced in countries with higher income inequality [12] where PA levels are lower in socioeconomically disadvantaged communities [9]. Therefore, it is vital to develop strategies to promote PA in individuals with various socioeconomic backgrounds, equitably as early as in preschool age.

The preschool setting presents unique opportunities to promote PA and health since the children are very young and habits can be more easily acquired. This makes this setting even more promising than the school setting. For example, preschool PA routines and practices can influence a major proportion of waking hours in children, since children in Sweden generally spend a large part of their waking hours at preschool. In addition, approximately 95% of Swedish 3–5-year-old children attend preschools regardless of their parent’s socioeconomic status (SES) [14]. Therefore, preschool interventions may offer each child an equitable opportunity in forming early healthy PA behaviors.

Moreover, preschools provide appropriate conditions to explore associations between PA, time spent outdoors and absence due to illness in children, which are important parameters from a public health as well as an economic perspective. For example, time spent outdoors at the preschool may contribute to health benefits through increased PA in children [15]. In addition, increasing time spent outdoors may reduce person-to-person transmission of infections, since children brought together in close environments, such as a preschool indoor environment, are mutually exposed to the transmission of pathogens [16]. It is possible that PA acts a mediator of the link between outdoor activities and absence due to illness in children [17]; reduced infection rates could be achieved by reducing transmission of disease as well as reducing host susceptibility through the beneficial health effects of PA. However, the association between outdoor stay and absence due to illness in children is currently unclear, as outdoor time has been shown to both reduce [18] and increase [19] absence due to illness in children. In addition, research has shown equivocal effects from PA on absence due to illness in children [20]. Nonetheless, it is conceivable that outdoor time impacts on infection rates by both decreasing the opportunity of transmission and by reinforcing immunity defense via PA. However, it is possible that outdoor activities also impact other morbidity (e.g., accidents and traumas); hence, the net effect may be concealed.

Previous meta-analyses and reviews of PA interventions in preschoolers indicate that interventions are likely to be effective when inducing environmental/organizational change in the preschool setting, targeting multicomponent factors such as integrated structured PA time, and ensuring unstructured outdoor time and instructions to preschool teachers on how to lead structured activity [21, 22]. Although policies may be potentially modifiable organizational factors in preschools, introducing preschool policy can be challenging since preschool policies often suffer from unclearness, poor implementation, and lack of tools for follow-up and measures of compliance [23, 24]. Furthermore, few previous studies have explored the association between preschool PA policies and children’s PA levels [25–27]. An observational study from Sweden has demonstrated higher PA levels in children enlisted in preschools with a PA policy [27]. This result is in line with findings from other countries [25, 26]. However, only a quarter of all participating preschools in the Swedish study had a PA policy [27]. In general, the few existing randomized controlled trials within the field suffer from small sample sizes and limited intervention duration [21, 22]. A systematic review by Wolfenden et al. focused on PA polices within preschool setting reported little benefit on PA outcomes [28]. This may be a result of the lack of an evidence-based policy, the policy not being adapted to the context, the absence of a theory base, and various challenges in implementation of the policy [28]. Similarly, a recent study by Carson et al. that employed a quasi-experimental pre-post design with accelerometer-based PA measurement also demonstrated no effect of preschool policy on PA outcomes of preschool children [29]. Accordingly, Wolfenden et al. emphasized the current scarcity of evidence and that well-design pragmatic randomized controlled trials with sufficient sample size and valid measurements are imperative to evaluate policy in a real-world setting [28]. To the best of our knowledge, there is only one ongoing pragmatic randomized trial testing preschool PA policy’s effect on PA levels of children [30]. In contrast to our trial which employ accelerometers to assess levels of PA, the ongoing trial by Nathan et al. use educator-reported PA [30]. Notably, although preschool teachers are key components in the implementation of policy, the policy’s effect on preschool teachers’ PA and its possible implications on parameters such as sick leave has not been studied previously. Such knowledge could add to the understanding of preschool PA policies and how they may affect change in PA. In addition, the equity perspective of PA interventions has been largely neglected previously [31].

### Objectives

The primary aim of the increasing ***C****hildren’s physical* ***A****ctivity by* ***P****olicy* (CAP) study is to evaluate the feasibility and effectiveness of implementing PA policies in preschools on preschool children’s PA levels. The secondary aims of the study include exploring the effectiveness of PA policy on preschool children’s (i) sedentary time, (ii) screen time, (iii) musculoskeletal fitness, (iv) sleep, (v) adiposity, (vi) psychosocial functioning, (vii) absence due to illness in children, and (viii) children’s PA opportunity outside preschool time such as active transport and organized sports participation as well as on preschool teachers’ (i) physical activity levels, (ii) sedentary time, (iii) sleep, (iv) adiposity, and (v) sick leave in teachers. We will also investigate the implementation of the policy and how this policy can be normalized into preschool teachers’ regular practice by exploring (i) dose and fidelity of the implementation, (ii) facilitators and barriers in implementing the preschool policy, and (iii) sustainability of the policy.

### Hypothesis

The hypothesis is that implementing PA policies in preschools will increase PA in young children and preschool teachers equitably across districts with diverse SES. We further hypothesize that implementing PA policies will lead to improvements in children’s musculoskeletal fitness, psychosocial functioning, sleep and reduce sedentary time, screen time, and absence due to illness in children and sick leave in preschool teachers. The policy may also influence preschool children’s PA opportunities outside preschool time such as increases in active transport. However, adiposity is hypothesized to not be affected as meta-analysis data showed limited evidence for an association between PA and adiposity among preschool children [32].

## Methods/design

### Study design and setting

The current study is a pragmatic cluster-randomized controlled superiority trial with two conditions (intervention and control) with preschools as clusters and the unit of randomization. A cluster randomization design was chosen because the intervention targets policy on the organizational level in preschools and preschools are natural clusters. Units of observations are preschool children and preschool teachers. The report of this trial protocol is guided by SPIRIT reporting guidelines [33] and we provide the detailed SPIRIT Checklist (see SPIRIT Checklist). We will report the findings of the trial according to guidelines outlined in the CONSORT extension for cluster-randomized trials [34]. The current study is being conducted in the Stockholm region of Sweden. To date in the Stockholm region, there are 30,969 children (1–5 years old) enrolled in a total of 539 public preschools (personal communication with Stockholm region representative, 2020-06-15). The Stockholm region can be divided geographically and administratively into 13 districts which vary in size, number of preschools, children, and resources.

### Ethical approvals and informed consent

Ethical approval has been obtained from the Swedish Ethical Review Authority (Dnr. 2020-03002), and this study has also been pre-registered at a database for clinical studies (https://clinicaltrials.gov) with reference number: NCT04569578. All items from the World health Organization Trial Registration Data Set can be found ClinicalTrials.gov. The registration of the study was completed before the recruitment of participants. The project coordinator contacted participating preschool staff about project information and provide information to distribute to the potential participating children and parents (Additional file 1). The project rationale, process, and motivational/explanatory videos are also available from the project website (Website in Swedish Rörelseprojektet (regionstockholm.se)). An informed consent form can be downloaded directly from this project website and physical copies of the informed consent form are available at the respective preschool. Written informed consent signed by parents of the participating children and participating preschool teachers will be gathered at the respective preschools. The informed consent will then be collected by one of the trained field workers prior to the baseline measurement. After collection, data in the informed consent will be manually typed into a database and stored securely on servers within the Center for Epidemiology and Community Medicine, Stockholm County Health Care Area (SLSO).

### Sample size

The required sample size to obtain a statistical power of 0.90 was projected with a change in MVPA as the main outcome variable and by using the means and standard deviations of the aforementioned observational preschool study assessing PA [27]. A 5-min change in MVPA has been suggested in previous meta-analysis to be a reasonable target of clinical importance [35]. Assuming an effect size of 5 min MVPA, an intra-preschool correlation of 0.2, a standard deviation of 25, coefficient of variation of 0.3 (average preschool size of 35 children 3–5 years), and approximately 2800 children (from 85 preschools) should be retained.

### Study population and recruitment

Based on other similar studies and the previously mentioned preschool study conducted by our research group [27], approximately 30% total non-participation is expected. Specifically, we expect an estimated accelerometer non-compliance rate of 10% (i.e., participants with insufficient wear time) and a dropout rate of 20% throughout the study period. Consequently, we aim to recruit approximately 4000 children aged 3–5 years from 100 public preschools within all 13 districts in the Stockholm region.

### Inclusion/exclusion criteria

**Fig. 1:**
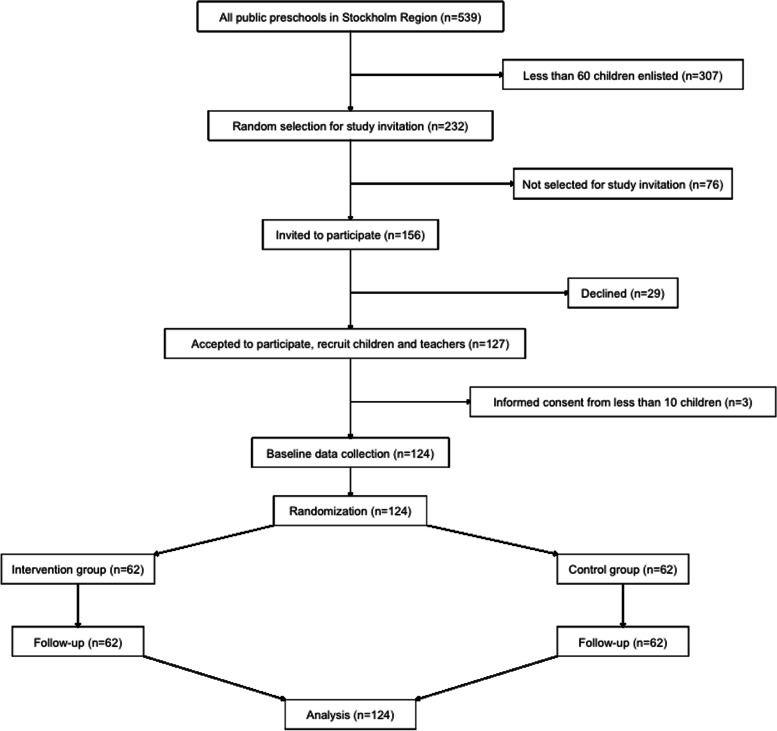
Flowchart of study recruitment, randomization, and follow-up

Figure 1 illustrates the recruitment of preschools and subsequent recruitment of preschool children and teachers based on the inclusion/exclusion criteria. Due to the logistical burden and time limit of the study, only preschools with more than 60 enlisted children are eligible for participation. Despite restricting the invitations to preschools with more than 60 children enlisted, we expect that approximately 35 children per school will elect to participate. Children in the participating schools might, for example, elect not to participate due to COVID-19 or time constraints—limiting the number of children per school (cluster size). In order to ensure a representative sample in each district, approximately 30% of all public schools with more than 60 enlisted children (all age groups) in each district will be randomly asked to participate in the study. The random selection of preschools for study invitation will be fulfilled by contacting preschool principals following a random order of invitation generated by Stata (Additional file 2. programming codes, random_invatation_preschool) until the number of preschools agreed to participate reached approximately 30% of all public preschools in that district. The recruitment of preschool children and teachers will be initiated provided that preschool principals agree to participate. All 3–5-year-old children enlisted at the participating preschools will be invited to participate and the participation will be confirmed by the informed consent of parents. In addition, full-time teachers at the participating preschools will also be invited to participate and provide informed consent. Preschools with less than 10 children with parental informed consent and children who are not able to able to take part in PA measures (e.g., mobility disability) will be excluded from participation. The limit of at least 10 children with parental informed consent per preschool was chosen due to logistical reasons to accommodate the accelerometer measurement burden in the study.

A total of 156 preschools were asked to participate in the study and 127 preschools (with median of 11 preschools per district) with 3775 children and 448 teachers agreed to participate. Apart from 3 preschools that collected less than 3 parental informed consents, 124 preschools participated in the baseline data collection. No preschools were lost to follow-up.

### Randomization and blinding

Preschools will be stratified by the 13 Region Stockholm districts because geographical, organizational, and resource factors in each district are likely to differ and influence the implementation and effectiveness of the policy. After baseline assessment, preschools will be randomly assigned to the intervention or control condition by 1:1 ratio within each of the 13 districts to ensure equal representation of intervention and control preschools. Block randomization with districts as blocks is conducted with randomizr package in R (Version3.6.1) (Additional file 3. programming codes: randomization_preschool). A single-blinded approach will be adopted with outcome assessor and data analyst blinded to the treatment allocation. Since the intervention is open-label, with only the outcome assessors and data analysts blinded, emergency unblinding for participants will not occur.

### Intervention description

The intervention components were developed based on the aforementioned observational preschool study [27] and systematic review data on the effectiveness of PA interventions for preschoolers, i.e., (i) organizational changes at the policy level, (ii) unstructured outdoor play, and (iii) instructions to preschool personnel on how to encourage child PA behaviors [21]. The intervention framework, a study website and delivery of information, has been established on the directional management level within all 13 districts within the Stockholm region and will be delivered directly to each intervention preschool after baseline measures and the randomization process. To increase parental engagement in the intervention group, participating parents will be provided with an information package (multi-lingual multi-media content) describing the intervention components, their importance, and practical information on how to engage in active transportation to/from preschool.

The intervention framework (on preschool level) includes:

i. Formalized PA policies:
  - A minimum of 3 h of total daily outdoor time, to be scheduled both in the morning and in the afternoon.
  - At least 10 min of teacher-led active play per day. An activity bank will be provided for inspirations of possible teacher-led activities.
  - A minimum of 1 weekly outdoor excursion which is commonly realized as a visit to local parks, playgrounds, or other recreational facilities. A map of recommendations of potential outdoor excursion areas will be provided.
  - Meet the children outdoors when they arrive at preschool and/or picked up by their parents.
  - Only use screen-based devices for educational teaching purposes.
  - Communicating to, and encouraging, the parents to actively transport their children to and from the preschool.
ii. A study website will provide information on the study process and measurements. The activity bank for inspiration of teacher-led active play and a map of recommendations for outdoor exertion, mentioned in PA policies b and c above, will also be accessible through the website. This study website may also serve as a platform for interprofessional education where preschool teachers can share and communicate about engagement in activities and their experience.
iii. Weekly follow-up and feedback by web-based questionnaires to determine fidelity and dose of intervention.

As the intervention is an organizational change allocated to the cluster (preschool) level, there will be no modification of the intervention from the individual (participant) level. There are no special criteria for discontinuing the intervention. The participant who wishes to discontinue the intervention have been instructed to immediately notify the research group.

### Intervention development and theory base

The interest for the intervention began when the previously mentioned observational study found a positive association between having a formalized PA policy in preschools and children’s PA levels [27], which attracted attention from politicians and stakeholders in the City of Stockholm. Realizing the potential of policy to increase PA among preschool children, the stakeholders took a formal political decision that all public preschools in the Stockholm region should implement a formalized PA policy. This decision is documented in the public preschools’ financial steering plan and implies that all public preschools in the Stockholm region should exert effort and financial resources in the implementation of a PA policy. In addition, a policy package should be developed and evaluated to provide a “model” for preschools to understand what PA policies to implement and effective strategies for implementation. The intervention policy package has been developed in co-creation among the research group, municipal childcare stakeholders, district officers from the 13 Stockholm region districts and a reference group consisting of preschool principals and teachers. The policy package has been discussed and acknowledged among all co-creators to increase the engagement, buy-in, feasibility, relevance, and sustainability of the intervention policy package as recommended by a previous meta-analysis on the effectiveness in co-creation of research [36]. The Behaviour Change wheel [37] was chosen as a theory base to understand and reflect on essential conditions (capacity, opportunity, and motivation) for behavior change in relation to the intervention’s functions and policy category. (The detailed logical model of theory of change for the policy package intervention based on behavioral change wheel can be found in Additional file 4 theory of change).

### Implementation of the intervention

This intervention is expected to scale up upon proof of effectiveness and feasibility. Therefore, no special implementation facilitation will be provided—to ensure external validity. Specifically, to ensure complete scalability, we will utilize a pragmatic approach where minimal additional resources are provided (no support beyond that of the intervention package), thereby evaluating whether the routine resources disposable to Swedish preschools are sufficient for the implementation of the intervention. The implementation of the policy package will be examined in detail following the process evaluation framework by Moore et al. [38]. To which extent the policy package is implemented (dose and fidelity) is followed up weekly by web-based questionnaires. Implementing the intervention policy package will not require alteration to usual preschool care pathways. More details of process evaluation are illustrated in the “Outcomes and measures” section.

### Control condition

Preschools randomized to the control condition are not provided with the intervention package and are encouraged to keep their usual practice and routines during the intervention period. However, in this pragmatic trial, control preschools are also under the influence of financial steering document, and they are free to implement any PA policy. In addition, communication between teachers from intervention and control groups and the study website may contribute to contamination. Therefore, PA policies in control preschools will be monitored at baseline, mid-point (3 months), and endpoint (6 months) to capture possible contamination effects and understand the possible change in their PA policy throughout the intervention.

### Outcomes and measures

In the present study, several instruments are used to assess outcomes in children and teachers. An additional file provides an illustration of all the instruments used, corresponding to the measured outcome variable, time point of measurement, and group relevance (i.e., measurement in both intervention and control groups or intervention group only) in detail (See Additional file 5). Questionnaires will be distributed via email to each respondent through Webropool 3.0. All questionnaires used can be found in Supplementary material 3 questionnaires. Questionnaires answered by preschool personnel are distributed only in Swedish. The questionnaires distributed to parents are available in Swedish, English, Arabic, Turkish, Somali, Tigrinya, and Swahili to cater to the need of parents with language barriers as an effort to retain a study sample containing individuals from different countries of origins. The questionnaires included as additional files (see Additional files 6, 7 and 8) are only available in English and questionnaires in other languages can be made available upon reasonable request.

### Physical activity levels (primary outcome) and sedentary time (secondary outcome) in children

PA levels in children such as total PA, MVPA, light PA (LPA), ST, and steps will be measured objectively using triaxial GT3X+ accelerometers, which have been tested extensively for reliability and validity in both pediatric and adult populations [39]. Children will be instructed to wear the accelerometer, on the non-dominant wrist, for 24 h during 7 consecutive days except during water activities (e.g., when showering and swimming) at both baseline and 6 months follow-up. The non-dominant hand will be predetermined by parents (information collected together with informed consent). Setup and analysis of accelerometer data will follow validated age-specific criteria and recommendations [39]. The accelerometer data sampling frequency is 30 Hz and data will be analyzed at 1-s epochs which suits the sporadic movement pattern of preschool children [39]. Raw accelerometer data will be downloaded through Actilife and analyzed using the GGIR package [40]. The classification of PA intensities and ST will follow the cut-offs developed for children with wrist-worn Actigraph GT3X+ by Hildebrand et al. [41, 42]. Steps will be determined using the Verisense step algorithm, which is integrated in GGIR to determine steps for wrist-worn accelerometry [43]. Accelerometer data consisting of ≥ 10 h/day wear time with > 3 days of measurement days will be considered valid [44]. However, participants with less valid days will be included in sensitivity analysis.

Results will be reported in categories of PA during the preschool time, PA outside preschool time, and PA during the entire day. This is to explore how the preschool policy affects PA during the preschool time frame and to assess potential extended effect beyond the preschool time frame and compensatory behavior [45]. Change in MVPA will be emphasized since PA in higher intensity is suggested to have greater benefits for health outcomes [3].

### Musculoskeletal fitness in children (secondary outcome)

Musculoskeletal fitness, in terms of handgrip strength, will be measured by an analog dynamometer (TKK 5825, Grip-A, Takei, Tokyo, Japan) validated in preschool-aged children [46]. Measurement of handgrip strength will be conducted at baseline and at 6 months follow-up by trained field workers to the nearest 0.1 kg on both dominant and non-dominant hands.

### Psychosocial functioning of children (secondary outcome)

The psychosocial functioning of children will be assessed by a parental report of the Strength and Difficulty Questionnaire (SDQ) which has been used extensively worldwide and validated also in Swedish settings [47]. Parents will be asked to fill in the SDQ and sleep questionnaire at both baseline and 6 months follow-up. The SDQ questionnaire uses a 3-point Likert scale to measure the children’s psychosocial strength and problems summarized into an emotional symptoms scale, conduct problems scale, hyperactivity scale, peer problem scale, and prosocial scale. All scales except for the prosocial scale are then summarized into a total difficulties score. The result for psychosocial functioning will be reported as the total difficulties score as well as separate scores on each of the scales mentioned above.

### Sleep of children (secondary outcome)

Sleep will be measured using a combination of 24 h accelerometry, a sleep time diary during periods of accelerometer measurements, and a parental questionnaire to reflect sleep quality during the past 6 months. All measurements for sleep will be conducted at baseline and 6 months follow-up. Sleep onset, sleep duration, and waking up will be determined by algorithms integrated in GGIR [48]. Appropriate algorithms for detection of sleep in children will be used. The sleep time diary included in the parental measurement week questionnaire consists of two questions regarding children’s wake up time and bedtime. In addition, parents will answer questions in SDQ and sleep questionnaire about children’s sleep duration and quality during the past 6 months and these questions are adapted from the validated Ages and Stages Questionnaire [49]. To minimize the burden of answering different questionnaires, the sleep quality questions were added to the SDQ questionnaire; therefore, the questionnaire is named SDQ and sleep questionnaire.

### Screen time in children (secondary outcome)

Children’s screen time, in minutes of screen time per day, will be reported by parents at baseline and endpoint during periods of accelerometer measurements in the parental measurement week questionnaire.

### Absence due to illness in children (secondary outcome)

Absence due to illness frequency and duration from 12 months prior to the intervention baseline and during the 6-month intervention period (18 months in total) will be collected from the City of Stockholm central preschool absence database, a database to which all parents of preschool children in Stockholm report all sick leave.

### Adiposity of children (secondary outcome)

Weight, height, and waist circumference will be measured, by trained field workers, at baseline and 6 months follow-up. All measurements will be conducted twice, and each measurement result will be recorded to the nearest 0.1 of the respective unit. Weight will be measured by validated scales (calibrated scale: VB2-200-EC, Vetek AB, Väddö, Sweden), and height will be measured by stadiometers (portable stadiometer: Seca 213, Seca, Chino, CA, USA). Weight and height will then be used to calculate body mass index (kg/m^2^), and children will be classified as normal weight, overweight, or obese based on body mass index criteria developed by Cole et al. [50]. Waist circumference will be measured at the level of the navel directly on the skin [51]. Due to the COVID-19 pandemic, field researchers are required to conduct the measurements outdoors; thus, the waist circumference measurement may be conducted over thin clothing in cold weather.

### Demographic information, children, and parents (descriptive)

Information on the age and sex of parents of the participating children will be derived from the personal registry number provided upon the consent of participation. Parents will also, at baseline, be asked to complete a descriptive information questionnaire including questions regarding their education level, occupation, country of birth, height, and weight.

### Children’s PA opportunities outside preschool time (process outcome)

Children’s PA opportunities including participation in organized sports and active transport to/from preschool will be assessed via parental measurement week questionnaire (during the weeks of accelerometer measures at baseline and 6 months follow-up). Parents will be asked about the time, duration and type of organized sports, and mode of transportation to/from preschool. In addition, parents will also answer an active transport questionnaire at baseline and 6 months follow-up regarding children’s general frequency of active transport, mode of transport, parental perception of the distance, and condition of transportation route during the past 6 months. Moreover, several parental practices (outdoor time during the weekend, parental perception of PA, parental PA levels, and active play with parents) which could potentially influence children’s daily PA levels will also be assessed by questions included in the parental questionnaire.

### Parental evaluation of the project and interview (process outcome)

A parental evaluation questionnaire will be distributed to parents of all participating children at 6 months follow-up. This evaluation questionnaire includes the parental rating (5 score scale) and open-ended questions on information quality, communication and perception of study influence, and experiences of study participation. Parents have the opportunity to report any unintended adverse effects of the intervention in this instrument. These questions will be used in qualitative analysis of potential facilitators and barriers of the study. At the end of this questionnaire, parents will be asked whether they want to participate in an interview to further explore the experience, influence, and potential of the study.

### Physical activity levels, sedentary time, and sleep in preschool teachers (secondary outcome)

Similar to children’s PA levels, ST, and sleep, these variables in teachers will also be measured objectively using triaxial GT3X+ accelerometers [39]. Preschool teachers will wear the accelerometer, on the non-dominant wrist, for 24 h during 7 consecutive days at baseline and 6 months follow-up. Minutes in total PA, MVPA, LPA, ST, and total steps during the preschool time, outside preschool time, and during the whole day will be reported for teachers. Sleep in preschool teachers will also be analyzed using 24-h accelerometry. Setup and analysis of accelerometer data will be performed following validated age-specific criteria and recommendations [39].

### Musculoskeletal fitness of preschool teachers (secondary outcome)

The method to assess musculoskeletal fitness in children will be used also for preschool teachers. Musculoskeletal fitness in terms of handgrip strength will be measured by an analog dynamometer (TKK 5825, Grip-A, Takei, Tokyo, Japan). Measurement of handgrip strength will be conducted at baseline and at 6 months follow-up by trained field workers and recorded to the nearest 0.1 kg on both dominant and non-dominant hands.

### Sick leave in preschool teachers (secondary outcome)

Sick leave frequency and duration from 12 months prior to the intervention baseline and during the 6-month intervention period (18 months in total) will be collected from the City of Stockholm central preschool absence database.

### Adiposity of preschool teachers (secondary outcome)

Weight and height will be measured by validated scales (calibrated scale: VB2-200-EC, Vetek AB, Väddö, Sweden) and stadiometers (portable stadiometer: Seca 213, Seca, Chino, CA, USA), and waist circumference will be measured at the level of the navel directly on the skin [51]. Due to the COVID-19 pandemic, field researchers are required to conduct the measurements outdoors; thus, the waist circumference measurement may be conducted over thin clothing in cold weather.

### Demography of preschool teachers (descriptive)

The age and sex of the preschool teachers will be derived from their personal registry number provided upon consent to participate in the study.

### Preschool policy and environmental characteristics (process outcome)

Preschool environmental/organizational characteristics such as available play equipment, playground characteristics, current policies, and working practices will be assessed by the validated Environment and Policy Evaluation and Observation as a Self-Report Instrument (EPAO-SR) [52]. The full EPAO-SR contains both PA and nutrition components in three surveys: Staff General, Director General, and Staff Daily respectively. A combination of Staff General and Director General surveys with only PA subscales will be used in the current study to access the classroom physical environment such as the amount of space for active play, preschool’s physical environment in terms of size and features of the preschool playground and routines, as well as policy regarding physical activity. The preschool playground characteristics and size will be double-checked and measured objectively via spatial mapping using publicly available satellite imagery (e.g., Google maps). Preschool-level routine screen time will be reported by preschool teachers in intervention preschools weekly through the intervention weekly follow-up questionnaire and via the validated EPAO-SR. [52] The use of EPAO-SR is important to identify what policies/practices that are in place prior to the intervention, and to assess if any other preschool environmental/organizational changes take place during the intervention period. It is also a crucial tool to measure the extent of any contamination by the intervention to the control preschools.

### Implementation of the policy package (process outcome)

Fidelity and dose of the implemented policy package will be documented through a weekly follow-up questionnaire, answered by preschool staff, throughout the intervention period. The extent to which the intervention can be normalized into preschool staff’s regular practice will be assessed through the validated [53] No-MAD questionnaire (an instrument for assessing implementation work based on normalization process theory) [54] at 6 months follow-up.

### Preschool teachers’ evaluation of the project (process outcome)

Communication, acceptance, facilitators, and barriers for implementation will be assessed through teachers’ evaluation questionnaires provided by all participating preschool teachers. At the end of the teachers’ evaluation questionnaire, preschool teachers will be asked whether they are willing to participate in focus groups and interviews. Focus groups and interviews with preschool personnel will also be undertaken post-intervention to collect more in-depth perspectives regarding the adequacy of the intervention, the feasibility of implementation, and suggestions for improvements. These qualitative discussions will generate rich data regarding the logistical challenges and the strengths and weaknesses of the intervention that could be missed in questionnaires. An experienced moderator, using a semi-structured interview guide, will facilitate the discussions. Sample questions will include:

- *What challenges did you experience when implementing the intervention?*
- *What solutions did you undertake to deal with these challenges?*
- *What characteristics of the intervention do you feel were most appropriate for increasing PA participation among preschoolers?*

Focus groups and interviews will be audio-recorded and transcribed verbatim.

### Data collection team and training

Five to seven field workers will be trained to assist in the data collection. They will be trained by the corresponding author Chu Chen, who has knowledge and experience with data collection for the project. The training will be conducted in two 4-h sessions with practice. The Project Coordinator will contact the preschools to schedule the data collection. The trained field workers will be paired into teams to assist each other in data collection. Chu Chen will also engage in the data collection and will be teamed with each field worker at least once to ensure the data collection quality. Chu Chen will always be available via phone should there be any questions during the data collection process.

### Data collection and data management

Participants will be asked to wear an accelerometer for 7 consecutive days for measurement of physical activity at both baseline and the 6-month follow-up. The accelerometer will be distributed at the respective participating preschool. The participants’ height, weight, waist circumference, and grip strength will be measured by trained field workers at the time the accelerometer is distributed. These measurements will be conducted twice to ensure data quality. After the data collection, the above measurements will be manually typed into a database on servers within the Center for Epidemiology and Community Medicine, Stockholm County Health Care Area (SLSO). All data will be typed in by one field worker and double-checked by a different field worker. Chu Chen will perform range checks for the collected data.

All questionnaires are digitized. Participants will receive a unique link for the questionnaires in the email address they provided in the informed consent. The questionnaires are collected through Webropol, which is a trustworthy tool used in Region Stockholm.

### Data retention plan

The project coordinator will keep the preschools informed of the trial status and reminds participants of follow-up. If participants missed measurement/follow-up, more chances are to be provided as much as resources and time allow. However, as clearly stated in the informed consent, participants have the right to opt-out of the study at any time without providing further reasons, no more data will be collected for participants who wish to discontinue the study.

### Confidentiality of data

All data will be pseudo-anonymized and presented at an aggregated level, which means that it is not possible to report the results to a specific preschool or child. All data is stored securely on servers within the Center for Epidemiology and Community Medicine, Stockholm County Health Care Area (SLSO). No unauthorized person can access the stored data. All personal data will be processed in accordance with the EU General Data Protection Regulation (GDPR). The Center for Epidemiology and Community Medicine (CES) is responsible for personal data, and SLSO is the data protection officer.

### Result dissemination

The results of the study will be disseminated through a series of workshops given to Childcare stakeholders and preschool staff. Several publications, both as reports issued by Region Stockholm and scientific literature in academic journals, will be written in order to disseminate the knowledge to different target groups.

### Statistical analysis

The baseline demographic characteristics of the participants will be summarized descriptively. Dropout participants’ characteristics will be explored by comparison to the non-dropouts. Fidelity and dose of implementation will be summarized as a score (fidelity × number of weeks of compliance). The change in PA levels and secondary outcomes, from pre- to post-intervention, will be analyzed with linear mixed-effect models (to allow both fixed and random effects) nested on the preschool level. The linear mixed-effects model can test several aspects of the study effects, e.g., effects over time inside a group, and groups against each other at a given time (e.g., intervention vs. control at 6-month follow-up). Subgroup analyses will be conducted to analyze the effect of the intervention in relation to socioeconomic aspects. Furthermore, we aim to explore if the COVID-19 pandemic has modified any implementation aspects of the intervention (via questionnaires on how various routines including outdoor time, hygiene aspects at preschools, homestay, and transportation mode to/from preschool have changed following the COVID-19 pandemic).

### Timeplan of the project

**Fig 2:**
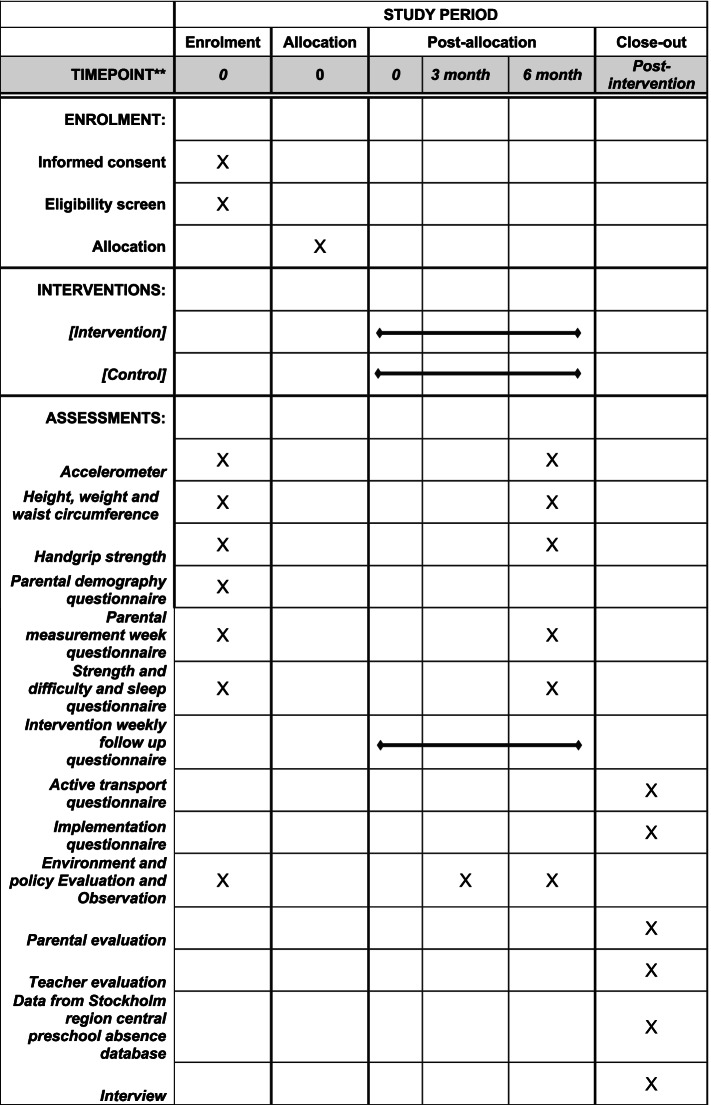
SPIRIT figure for schedule of enrollment, intervention, and assessment

Figure 2 illustrates the schedule of enrollment, interventions, and assessments according to SPIRIT guidance.

## Discussion

The CAP study aims to evaluate the feasibility and effectiveness of implementing a formalized policy package in Stockholm public preschools to increase levels of PA in preschool children. This is an unprecedented co-creation project among the City of Stockholm, childcare stakeholders, preschool staff, and the research group with the potential to finally impact levels of PA in more than 30,000 preschoolers, in more than 500 preschools in the Stockholm area. The project has received media and political attention which has led to a formal political decision that all 539 public preschools within the Stockholm region should implement a formalized PA policy (included in the public preschool financial steering document). To ensure evidence-based policymaking, it is imperative to evaluate the feasibility and effectiveness of this PA policy implementation. To the best of our knowledge, there is no other large-scale pragmatic cluster-randomized intervention study on preschool organizational level, evaluated with robust instruments in Sweden or elsewhere. This project will contribute to the implementation of PA policies at the organizational level of preschools, by providing reliable evidence of its effect on objectively measured PA and health markers in children, through an equity perspective.

The main strengths of this study include the pragmatic cluster-randomized controlled study design and a large sample size, particularly for preschool-aged children who imperatively need scalable effective interventions to improve their PA. In addition, the co-creation of the policy package among the research group, municipal childcare stakeholders, district officers from the 13 Stockholm region districts, and a reference group consisting of preschool directors and preschool teachers will likely increase the buy-in, feasibility, relevance, and sustainability of the intervention policy [36]. Moreover, this study is a pragmatic trial without implementation facilitation and with the control group observed in “usual conditions,” i.e., left “free” to implement any policy. This kind of pragmatic approach evaluates the policy package in a real-life setting and provides insight into the effectiveness of a co-created policy package in increasing PA levels in preschool children. This will provide valuable information for decision-makers regarding feasibility, effectiveness, and the conditions for scaling up. Another major strength of the present study is the objectively measured PA, which increases reliability and validity of data compared to subjective assessment [39]. Finally, in order to generalize the results to a broader population and address the lack of equity perspective in previous studies, the present study covers multiple districts with variations in socioeconomic status, and the study information and questionnaires have been translated into most of the major languages spoken in Sweden.

There are several potential limitations of the planned study. First, the PA policy initiative, included in the preschool financial steering document, was launched before the baseline measurement. Hence, the baseline measurement may be affected by this initiative. Second, although only intervention preschools are provided with the intervention material, contamination may occur since the communication between staff from different preschools cannot be excluded. However, the policy in place and preschool practices will be measured closely in both intervention and control preschools at baseline, mid-point, and endpoint of the study to monitor and understand the effect of an active control group and possible contamination. The effects of the intervention may be underestimated due to these limitations, but a detailed understanding of these limitations give insights regarding the effectiveness of a policy intervention under real-life conditions. Third, the ongoing COVID-19 pandemic and accompanying societal strategies (e.g., social distancing recommendations) to limit the spread of COVID-19 has brought restrictions on daily living and changes in preschool routines. More specifically, preschools in Sweden are recommended to increase the outdoor time during preschool hours. This change is likely to influence the study results through increased PA since extended outdoor time is an important component of the intervention. Nevertheless, it is hard to perceive and predict the future development of COVID-19 and its potential influence during the course of the study. To capture potential COVID-19 pandemic effects on intervention components (e.g., time spent outdoors), web-based questionnaires will be delivered to both participating control and intervention preschool teachers and parents. The questionnaires will contain questions on how various routines including outdoor time, hygiene aspects at preschools, homestay, and transportation mode to/from preschool have changed following the COVID-19 pandemic. It is also possible that the COVID-19 pandemic has increased awareness of the importance of health, PA, and outdoor time in the general population. Thus, the preschool personnel may be more motivated to implement and sustain the policy. These potential effects will be captured by program evaluation questionnaires and interviews.

This study will provide robust evidence of the effect of organizational changes in preschools on children’s PA and indicators of health. Notably, such robust evidence is urgently warranted as Swedish preschool children’s PA levels are currently low, which may impact their long-term health. Finally, as the intervention has been developed in co-creation with the City of Stockholm, it has the possibility to, if proven effective, be implemented rapidly within preschools.

## Trial status

The trial was prospectively registered at clinicaltrials.gov with reference number NCT04569578. That is, the trial protocol was finalized and published before any recruitment or data collection took place. By the time of submission to *Trials*, the recruitment of the participants had been completed (recruitment period: 2020-09-23- 2021-07-02) and the data collection/study visit was ongoing. We were unable to submit the protocol to Trials before recruitment as the COVID-19 pandemic drastically increased the resources required to recruit participants, resulting in a delay of submission. Important changes to the protocol, if any, will be communicated to the journal and trial registry. Until May 2023, no change has been made to the trial protocol as pre-registered at ClinicalTrials.gov.

## Supplementary Information

**Additional file 1.**

**Additional file 2.**

**Additional file 3.**

**Additional file 4.**

**Additional file 5.**

**Additional file 6.**

**Additional file 7.**

**Additional file 8.**

**Additional file 9.**

## Data Availability

There is no data included in this study protocol. The data collected in this study will be stored in the central database in Centre for Epidemiology and Community Medicine (CES). In the future publication regarding trial results, we aim for open access for the public access of knowledge and all statistical codes will be made available. Dataset cannot be made publicly available due to restriction in ethical approval unless additional approval is granted for reasonable request by the ethical review committee.

## Abbreviations

PA: Physical activity
MVPA: Moderate to vigorous physical activity
ST: Sedentary time
SES: Socioeconomic status
CAP: Increasing Children’s physical Activity by Policy study
SDQ: Strength and Difficulty Questionnaire
EPAO-SR: Environment and Policy Evaluation and Observation as a Self-Report Instrument
