# Supplementary material for: Increasing Children’s physical Activity by Policy (CAP) in preschools within the Stockholm region: study protocol for a pragmatic cluster-randomized controlled trial
Source: Trials. 2022 Jul 19;23:577. doi: 10.1186/s13063-022-06513-4 (PMC9295109; doi:10.1186/s13063-022-06513-4)
Supplement: Supplementary file 1 — Additional file 1. [file 13063_2022_6513_MOESM1_ESM.pdf]

Research person information

Increasing Childrens`s physical Activity by Policy (CAP) in Stockholm County preschools

Information to guardians

What is the project about and why do you want us to participate?

Both in Sweden and internationally, studies show that a large proportion of children, already in preschool age, are not meeting the World Health Organisation (WHO) recommendation of at least 60 minutes of moderate to vigorous intensity physical activity per day. The purpose of the recommendation is to create conditions to fullfil all children's right to good physical and mental health. Several studies show that girls' physical activity levels are lower than that of boys. It is important to create conditions for physical activity that lead to both boys and girls achieving the recommendation. The Centre for Epidemiology and Community Medicine (CES), in collaboration with the City of Stockholm's municipal preschools, will in this project measure physical activity levels of approximately 3000 children aged 3-5 years. The aim of the project is to investigate whether a strategy for physical activity in preschool contributes to an increase in children's physical activity levels. The project will encourage moderate to vigorous intensity physical activity such as spontaneous play, walking and teacher-led play, etc. Your childs' preschool is one of the participating preschools.

How is the project carried out?

As a participating guardian, you will initially answer a questionnaire concerning the child's mental health, as well as some questions related to sleep. You will also answer a short questionnaire concerning your level of education, occupation and othe lifestyle related aspects. The child's height, weight, waist circumference and grip strength will be measured, and these measurements will be taken at the start-up day for the physical activity measurement at the child's preschool. The physical activity levels are measured over seven subsequent days using an accelerometer; a small device worn on the child's non-dominat wrist. During the physical activity measure week you will receive a short daily questionnaire concerning the child's daily routines. After six months, a repetition of all measurements, as well as the daily questionnaire, will be made. All questionnaires are digitized. What is required of you as a guardian is to make sure that the child wears the accelerometer, preferably both day and night. Wearing the accelerometers at night provides a basis for analysis of possible relationships between sleep patterns and physical activity levels. The accelerometer is not waterproof; thus, it must be taken off when the child showers or swims.

Possible consequences and risks of participating in the project

There are no documented risks regarding the use of accelerometers. It may be that the child finds it uncomfortable to wear the device at night. If this is the case, you can decide within

the family whether it should be used at night. Body measurements and measures of grip strength do not pose any risks.

What happens to our data?

The project will collect and record the necessary information about you and your participating child/children. The child's absence, in addition to the information described above, will also be collected (from the Stockholm City register on preschool absence). All data in the project will be pseudoanonymized and presented at an aggregated level, which means that it is not possible to derive results to a specific preschool or child. All data is stored securely on servers within the Centre for Epidemiology and Community Medicine, Stockholm County Health Care Area (SLSO).

Your answers and results will be processed so that no unauthorized persons can access them. All personal data will be processed in accordance with the EU General Data Protection Regulation (GDPR). Centre for Epidemiology and Community Medicine (CES) is responsible for personal data, and SLSO is the data protection officer.

You have the right to request information about your stored personal data from Stockholm county's health care area every year. You also have the right to lodge a complaint with the Swedish Data Protection Authority if you believe that your personal data has been misprocessed.

The electronic material will be destroyed at the time the project is formally terminated.

Information about the results of the project

After completion of measurements and compilation of results, you will receive a report with detailed data on your child's physical activity patterns during the two measurement weeks. The report can be an important support in creating and/or maintaining conditions for an adequate level of physical activity for your child/children. Achieving recommended levels of physical activity is linked to major health benefits for the child, both presently and in the future.

Informed consent

Before participating in the project, you will fill in an informed consent where you agree to participate in the project. If you are two guardians, both need to agree to participation in the project. The consent is included at the end of this document.

Participation is voluntary

Your own and your child's participation is voluntary and you may choose to cancel participation at any time. If you choose not to participate or wish to cancel your participation, you do not have to state why. To cancel participation, contact the project manager.

Insurance and compensation

The research does not involve any known risks and thus injuries etc. are covered by the insurance cover that exists in the preschool/home insurance, etc. Participating children in both intervention and control preschools will receive a small gift in return for participation.

Responsible for the project

Center for Epidemiology and Community Medicine is the head of the project and data controller. Daniel Berglind the principal researcher and the contact person (contact information below).

Daniel Berglind | PhD | Associate Professor
Department of Global Public Health | Karolinska Institutet
Centre for Epidemiology and Community Medicine
104 31 Stockholm | Solnavägen 1E
+46 70-364 47 97
E-mail: daniel.berglind@ki.se
robert.berglind@sll.se

Consent to participate in the project

To guardians (if you are two guardians, both need to sign):
I/we have been informed about the project and have had the opportunity to ask questions.

☐ I/we agree to participate in the study "Increasing Childrens`s physical Activity by Policy (CAP) in Stockholm County preschools

☐ I/we agree that information about me/us is processed in the manner described in the research person information.

Child's name	Child's social security number (YYYYMMDD-XXXX)

Child's preschool	District (stadsdel)

Printed name guardian 1	Social security number guardian 1 (YYYYMMDD-XXXX)

E-mail address guardian 1

Printed name guardian 2	Social security number guardian 2 (YYYYMMDD-XXXX)

E-mail address guardian 2

Place and date	Signature guardian 1

Place and date	Signature guardian 2

Please provide the e-mail address to which the daily questionnaire during the physical activity measurement week should be sent. If you have several children participating in the project, each child needs to be linked to a unique email address. All questionnaires are pseudoanonymized, which means that the child's name is not stated in the questionnaire, and only one questionnaire is sent out to each email address.

Email address questionnaires

The accelerometer (physical activity device) should sit on the non-dominant hand:

The child is: ☐ Left-handed ☐ Right-handed
