# Supplementary figures and images for: Increasing Children’s physical Activity by Policy (CAP) in preschools within the Stockholm region: study protocol for a pragmatic cluster-randomized controlled trial
Source: Trials. 2022 Jul 19;23:577. doi: 10.1186/s13063-022-06513-4 (PMC9295109; doi:10.1186/s13063-022-06513-4)

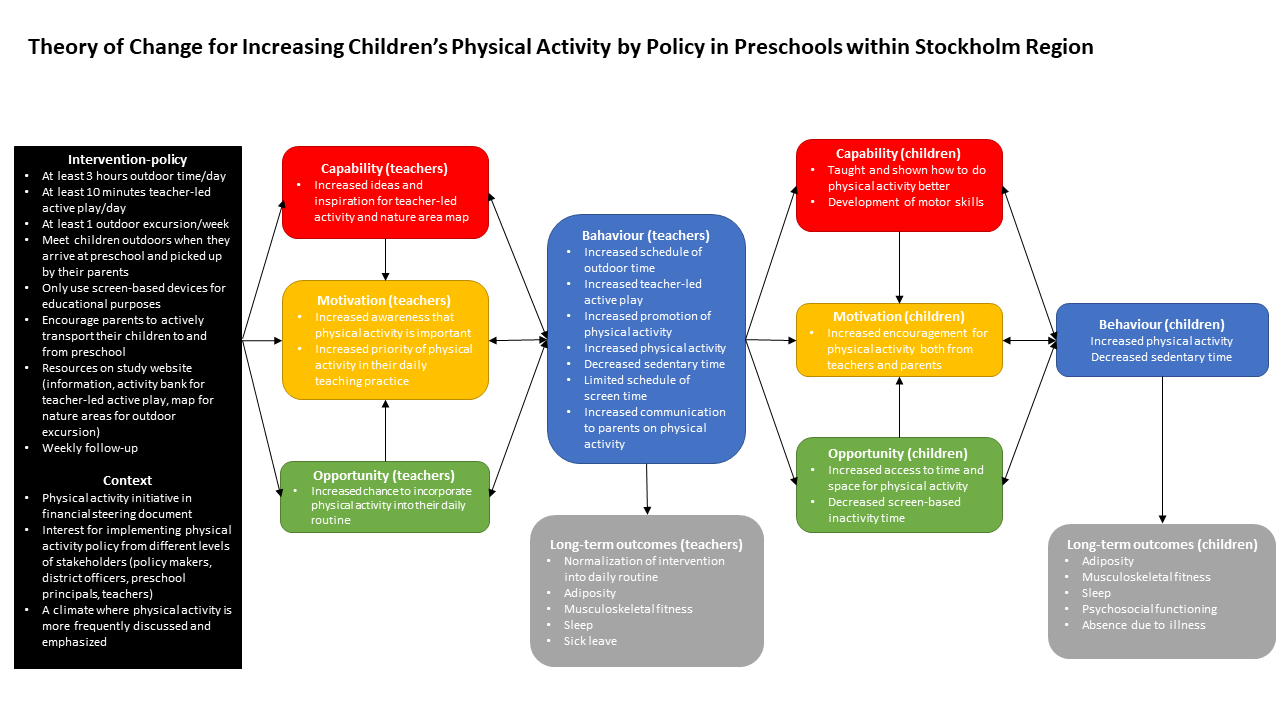

Supplement: Supplementary file 4 — Additional file 4. [file 13063_2022_6513_MOESM4_ESM.tif]
