# Supplementary material for: Increasing Children’s physical Activity by Policy (CAP) in preschools within the Stockholm region: study protocol for a pragmatic cluster-randomized controlled trial
Source: Trials. 2022 Jul 19;23:577. doi: 10.1186/s13063-022-06513-4 (PMC9295109; doi:10.1186/s13063-022-06513-4)
Supplement: Supplementary file 5 — Additional file 5. [file 13063_2022_6513_MOESM5_ESM.docx]

| **Additional file 5. Instruments, outcomes assessed and timepoint of assessment** | | | | | | | |
| --- | --- | --- | --- | --- | --- | --- | --- |
| **Instruments** | **Outcome assessed** | | | **Timepoint of assessment** | | | |
| **Objective assessment** | Measured in children | | Measured in teachers | Baseline | mid-point | End-point | Post-intervention |
| Accelerometer GT3X+ | Physical activity levels, sedentary time and sleep of children | | Physical activity levels, sedentary time and sleep of teachers | x |  | x |  |
| TKK 5825, Grip-A, Takei, Tokyo, Japan | Musculoskeletal fitness of children | | Musculoskeletal fitness of teachers | x |  | x |  |
| Portable stadiometer: Seca 213, Seca, Chino, CA, USA | Height of children, adiposity | | Height of teachers, adiposity | x |  | x |  |
| Calibrated scale: VB2-200-EC, Vetek AB, Väddö, Sweden | Weight of children, adiposity | | Weight of teachers, adiposity | x |  | x |  |
| Tape measure | Waist circumference of children, adiposity | | Waist circumference of teachers, adiposity | x |  | x |  |
|  |  | |  |  |  |  |  |
| **Questionnaires** | Answered by parents | | Answered by preschool teachers |  |  |  |  |
| Parental measurement week questionnaire | Sleep time,screen time, children’s physical activity opportunity outside preschool time | |  | x |  | x |  |
| SDQ^a^ and sleep questionaire | Phychosocial functioning and sleep quality of children | |  | x |  | x |  |
| Parental demography questionnaire | Descriptive demography information of parents | |  | x |  |  |  |
| Active transport parental questionnaire | Children’s PA opportunity outside preschool | |  | x |  | x |  |
| Parenal evaluation of the project | For process evaluation | |  |  |  | x |  |
| Intervention weekly follow-up |  | | Dose and fidelity of intervention* |  | x | x |  |
| Implementation (No-MAD)^b^ |  | | The implementation and normalization of intervention* |  |  | x |  |
| EPAO-SR^c^ |  | | Policy and other preschool characteristics* | x | x | x |  |
| Teachers' evaluation of project |  | | Process evaluation |  |  | x |  |
|  |  | |  |  |  |  |  |
| **Stockholm region central preschool abscense database** | Abscense due to illness in children | | Sick leave in teachers |  |  | x |  |
| **Interviews** | Process evaluation | | Process evaluation |  |  |  | x |
| *Preschool level outcomes; ^a^Strength and Difficulty Questionnaire; ^b^an instrument for assessing implementation work based on normalization process theory; ^c^Environment and Policy Evaluation and Observation as a Self-Report Instrument | | | | | | | |
| No background color: measured in both intervention and control groups | | Grey background color: measured only in intervention groups | | | | | |
