# Supplementary material for: Increasing Children’s physical Activity by Policy (CAP) in preschools within the Stockholm region: study protocol for a pragmatic cluster-randomized controlled trial
Source: Trials. 2022 Jul 19;23:577. doi: 10.1186/s13063-022-06513-4 (PMC9295109; doi:10.1186/s13063-022-06513-4)
Supplement: Supplementary file 6 — Additional file 6. [file 13063_2022_6513_MOESM6_ESM.pdf]

Basic information on parents

Increasing Children's physical Activity byPolicy (CAP): a study in Stockholm County Preschools

In this questionnaire you will be answering a few questions about yourself. All your personal data will be handled in accordance with EU's General Data Protection Regulation (GDPR). Data analysis and data presentation will be on group level. This means that the result of the study cannot be used to identify a specific preschool, or a specific child or parent.

1. What is your highest level of completed education?

- ◯ Elementary school/primary school
- ◯ 2 years of upper secondary school or professional school
- ◯ 3-4 years of upper secondary school
- ◯ University, less than 3 years
- ◯ University, more than 3 years

2. What is your current employment status?

You may choose more than one alternative

- ☐ Employed, please specify % of full time employment: ______
- ☐ Self employed
- ☐ On leave or parental leave
- ☐ Studying or doing an internship
- ☐ Part of a labour market policy program
- ☐ Looking for employment
- ☐ Retired
- ☐ On disability ("Sjuk- eller aktivitetsersättning")
- ☐ On sick leave
- ☐ Homemaker
- ☐ Other, please specify: ______

3. What is, or used to be, your main profession?

If you are currently not working, please describe what used to be your main profession or work. If you have several jobs, please fill in the one that is your primary job. Please try to write as detailed as possible. For example, write primary school teacher instead of teacher, bus driver instead of driver, etc.

4. What is your country of birth?

- ◯ Sweden
- ◯ Other, please specify:

The following questions focus on your leisure time, both together with your child and by yourself.

5. On a regular weekend, I am outside with my child:

- ☐ About 1 hour/day
- ☐ 1-2 hours/day
- ☐ 2-3 hours/day
- ☐ More than 3 hours/day
- ☐ We usually do not go outside on weekends

6.
When I am outside with my child on weekends:

Please select the answer that fits best

- ☐ I usually watch him/her play
- ☐ I usually play with him/her
- ☐ We usually do not go outside on weekends

7.
When I am outside with my child, we usually

You may choose more than one answer

- ☐ Go to a playground

- [ ] Go to a park/green area
- [ ] Go out into nature (for instance a forest or nature reserve)
- [ ] We usually do not go outside on weekends
- [ ] Other, please specify: ______

In the questions below, we make a distinction between ***physical activity*** at a moderate level and ***exercising*** at a more intense level. You can think of daily physical activity as something that you do every day; for instance walk or bike, clean the home, take the stairs instead of the elevator, have a physically demanding job. When you exercise at an intense level your heart rate increases above moderate, and it usually involves showering after

8. Please select the alternative that best describes your level of daily physical activity:

- ○ My level of daily exercise is much too low
- ○ A little more daily exercise would be good
- ○ My level of daily exercise is sufficient

9. Please select the alternative that best describes your level of exercise at a more intense level:

- ○ I exercise 1-2 times a month
- ○ I exercise once a week
- ○ I exercise 2-3 times a week
- ○ I exercise more than 3 times a week
- ○ I don´t exercise at all

10. What is your height?

- ○ Please answer in centimeters: ______
- ○ I don´t know/don´t want to answer

11. What is your weight?

- ○ Please answer in kg: ______
- ○ I don´t know/don´t want to answer
