# Supplementary material for: Increasing Children’s physical Activity by Policy (CAP) in preschools within the Stockholm region: study protocol for a pragmatic cluster-randomized controlled trial
Source: Trials. 2022 Jul 19;23:577. doi: 10.1186/s13063-022-06513-4 (PMC9295109; doi:10.1186/s13063-022-06513-4)
Supplement: Supplementary file 7 — Additional file 7. [file 13063_2022_6513_MOESM7_ESM.pdf]

Centrum för epidemiologi och samhällsmedicin
REGION STOCKHOLM

Mental health and sleep

In this questionnaire you will be answering questions regarding your child`s ability to concentrate, his or her behaviour, relations with friends and sleep.

SDQ (The Strengths and Difficulties Questionnaire)

Below are questions from SDQ (The Strengths and Difficulties Questionnaire). SDQ is a brief emotional and behavioural screening questionnaire for children and young people, and the form is based on experience of two other established and internationally well-spread instruments; Rutters and ChildBehavior Checklist (CBCL). The SDQ is one of the most widely and internationally used measurements of child mental health. The method consists of five parts: Hyperactivity/concentration, behaviour, friendships, emotional symptoms and prosocial behaviour (for instance caring and generosity).

Please choose the answer (not true, somewhat true, certainly true) that best applies to your child. It is of great value that you answer all questions, even if you´re not quite sure or find the question strange. The answers should reflect your child´s behaviour during the past 6 months.

	Not True	Somewhat True	Certainly True
Considerate of other people´s feelings	○	○	○
Restless, overactive, cannot stay still for long	○	○	○
Often complains of headaches, stomach-aches or sickness	○	○	○
Shares readily with other children (treats, toys, pencils etc.)	○	○	○
Often loses temper	○	○	○
Rather solitary, prefers to play alone	○	○	○
Generally well behaved, usually does what adults request	○	○	○
Many worries or often seems worried	○	○	○
Helpful if someone is hurt, upset or feeling ill	○	○	○

	Not True	Somewhat True	Certainly True
Constantly fidgeting or squirming	◯	◯	◯
Has at least one good friend	◯	◯	◯
Often fights with other children or bullies them	◯	◯	◯
Often unhappy, depressed or tearful	◯	◯	◯
Generally liked by other children	◯	◯	◯
Easily distracted, concentration wanders	◯	◯	◯
Nervous or clingy in new situations, easily loses confidence	◯	◯	◯
Kind to younger children	◯	◯	◯
Often argumentative with adults	◯	◯	◯
Picked on or bullied by other children	◯	◯	◯
Often offers to help others (parents, teachers, other children)	◯	◯	◯
Can stop and think things out before acting	◯	◯	◯
Can be spiteful to others	◯	◯	◯
Gets along better with adults than with other children	◯	◯	◯
Many fears, easily scared	◯	◯	◯
Good attention span, sees work through to the end	◯	◯	◯

Do you have other comments or concerns that you would like to share?

All in all, do you think your child has difficulties at one or more of these areas; with feelings, koncentration, behaviour or being with others?

	No	Small difficulties	Obvious difficulties	Serious difficulties
	○	○	○	○

For how long have these difficulties existed?

	Less than a month	1-5 months	6-12 months	More than a year
	○	○	○	○

Is your child worried about or suffering from his or her difficulties?

	Not at all	Just a little	Quite much	A lot
	○	○	○	○

Do these difficulties interfere with the childs everyday life on any of the following areas?

	Not at all	Just a little	Quite much	A lot
At home	○	○	○	○
With friends	○	○	○	○
In learning situations	○	○	○	○

	Not at all	Just a little	Quite much	A lot
In leisure time activity	○	○	○	○

Are these difficulties a burden for you or for the family?

	Not at all	Just a little	Quite much	A lot
	○	○	○	○

Below are questions regarding your child´s sleep.

Does your child have trouble falling asleep in the evening?

	Often	Sometimes	Rarely	Never
	○	○	○	○

Does your child sleep at least 10 hours a day?

	Often	Sometimes	Rarely	Never
	○	○	○	○

Does your child wake up 3 or more times a night?

	Often	Sometimes	Rarely	Never
	○	○	○	○

Do you consider that your child has difficulties daytime due to poor sleep?

	Not at all	Just a little	Quite much	A lot
	○	○	○	○
