# Supplementary material for: Increasing Children’s physical Activity by Policy (CAP) in preschools within the Stockholm region: study protocol for a pragmatic cluster-randomized controlled trial
Source: Trials. 2022 Jul 19;23:577. doi: 10.1186/s13063-022-06513-4 (PMC9295109; doi:10.1186/s13063-022-06513-4)
Supplement: Supplementary file 8 — Additional file 8. [file 13063_2022_6513_MOESM8_ESM.pdf]

Parents evaluation of the study

Thank you, to you and your child, for participating in "Increasing Children's physical Activity by Policy (CAP): A study in Stockholm county preschools" during the autumn of 2020 and spring of 2021. Your contribution is valuable in finding out how preschools can benefit from a strategy of promoting children's physical activity. We hope that you have been inspired, and maybe even gained knowledge about the importance of physical activity for your child's physical, mental and cognitive health. We would like to know how you have experienced the study. Please answer the questions below. Thank you for your help!

1. Information about the study, distributed by the preschool was easy to understand (for instance initially through weekletters and the consent form)

	1	2	3	4	5	
Not at all	◯	◯	◯	◯	◯	Absolutely

2. If you answered 1, 2 or 3 in the question above, please indicatewhat did not work:

3. Information about the study, distributed by Center for Epidemiology and Community Medicine (CES), was easy to understand (for instance questionnaires and email)

	1	2	3	4	5	
Not at all	◯	◯	◯	◯	◯	Absolutely

4. If you answered 1, 2 or 3 in the question above, please indicate what did not work:

5. Information on the study's website was clear and informative (text and films)

	1	2	3	4	5	
Not at all	○	○	○	○	○	Absolutely

6. If you answered 1, 2 or 3 in the question above, please indicate what did not work:

7. When I had questions about the study, I turned to ___ for information:

Multiple choices can be market

- ☐ Principal/deputy principal in my child`s preschool
- ☐ Teachers in my child`s preschool
- ☐ Project leader from CES
- ☐ Researcher from CES

8. When I had questions, I got answers/information quickly

	1	2	3	4	5	
Not at all	○	○	○	○	○	Absolutely

9.

If you answered 1, 2 or 3 in the question above, please indicate what did not work:

10. The questionnaires, before and during the course of the study, contained relevant questions

	1	2	3	4	5	
Not at all	○	○	○	○	○	Absolutely

11. If you answered 1, 2 or 3 in the question above, please indicate howthe questions lacked relevance:

12. My child wore the accelerometer:

- ○ Both day and night
- ○ Only during daytime

13. My child thought the accelerometer was:

- ○ Fun to wear
- ○ Ok to wear
- ○ Difficult or annoying to wear, please specify how:

14. Have the study influenced your behaviour related to your child`s physical activity:

Multiple choices can be marked

- ☐ No

- [ ] Yes, we walk/bike more often to and from preschool
- [ ] Yes, we go outside more during the weekends
- [ ] Yes, I think more about the importance of my child´s physical activity
- [ ] Yes, I have a higher level of physical activity myself now, compared to before the study
- [ ] Other, please specify: ______

15. Have you noticed any difference in your child that can be related to the study?

Multiple choices can be marked

- [ ] No
- [ ] My child sleeps better
- [ ] My child eats better
- [ ] My child is more energetic
- [ ] Other, please describe: ______

16. For each statement, please mark the alternative that best describes your experience.
1: Not at all, 5: Absolutely

	1	2	3	4	5
Participating in the study was interesting	○	○	○	○	○
The study is important in improving children´s health	○	○	○	○	○
It was a lot of trouble participating in the study	○	○	○	○	○
Because of the study my child is now more physically active	○	○	○	○	○

17. What was the biggest challenge for you as a parent, in participating in the study?

______

18. If you were to specify something that was particularly good about the study, what would it be?

19. May we contact you for a follow up interview about the study?

The interview will be about 15 minutes long, either conducted through phone or in person. The goal of the interview is to gain a deeper understanding of your answers to the questions in this questionnaire.We greatly appreciate your willingness to participate in an interview.

- ◯ Yes
- ◯ No
